# Dietary Supplementation of *Lactobacillus johnsonii* RS-7 Improved Antioxidant and Immune Function of Weaned Piglets

**DOI:** 10.3390/ani13101595

**Published:** 2023-05-10

**Authors:** Kai Zhao, Heng Yin, Honglin Yan, Wenjie Tang, Hui Diao, Qi Wang, Renli Qi, Jingbo Liu

**Affiliations:** 1 School of Life Science and Engineering, Southwest University of Science and Technology, Mianyang 621010, China; 2 Yibin Municipal Bureau of Agriculture and Rural Affairs, Yibin 644000, China; 3 Livestock and Poultry Biological Products Key Laboratory of Sichuan Province, Sichuan Animtech Feed Co., Ltd., Chengdu 610066, China; 4 Chongqing Academy of Animal Science, Chongqing 402460, China

**Keywords:** *Lactobacillus johnsonii* RS-7, weaned piglets, immune, antioxidant

## Abstract

**Simple Summary:**

In this study, we investigated the immune and antioxidant properties of *Lactobacillus johnsonii* RS-7 in weaned pigs. We found that dietary supplementation of *Lactobacillus johnsonii* RS-7 can effectively improve growth performance, enhance immune function, and improve the antioxidant performance of weaned pigs. The results of this study provide a feasible nutritional means to solve the weaning stress of piglets effectively.

**Abstract:**

We investigated the effects of dietary supplementation of lactic acid bacteria on the immune and antioxidant performance of weaned pigs. A total of 128 Duroc × Landrace × Yorkshire piglets weaned on day 28 with an average body weight of 8.95 ± 1.15 kg were selected and randomly divided into four treatment groups according to body weight and sex for a 28-day study. The four dietary treatments were basal diet (CON), and CON with 0.05% (LJ0.05), 0.1% (LJ0.1), and 0.2% (LJ0.2) *Lactobacillus johnsonii* RS-7, respectively. The lowest feed-to-gain ratio (F:G) was found when LJ0.1 was added to the diet. The addition of compound lactic acid bacteria to the diet increased the concentrations of TP, ALB, IgA, and IgM on day 14 and IgG, IgA, and IgM on day 28 (*p* < 0.05) in the blood, with trait values greater for pigs fed LJ0.1 than CON pigs (*p* < 0.05). Concentrations of antioxidants (CAT, T-AOC, MDA, T-SOD, and GSH) in serum, intestinal mucosa, spleen, liver, and pancreas improved. In summary, dietary supplementation of *Lactobacillus johnsonii* RS-7 improved the antioxidant and immune function of weaned piglets.

## 1. Introduction

In recent years, with the concern about antibiotic residues in animal products and bacterial antibiotic resistance, there is an urgent need to develop and research safe and effective alternatives to antibiotics. Adding probiotics to replace antibiotics has received widespread attention [1]. Lactic acid bacteria (LAB), as a kind of common and safe probiotic, has also been widely discussed [2].

LAB belong to gram-positive bacteria, which are arranged in single, double, or short chains, mainly including Lactobacillus, Streptococcus, Leuconostoc, Bifidobacterium, Pediococcus, and Enterococcus [3,4]. Preliminary studies have shown that LAB can effectively improve growth performance, feed conversion efficiency, nutrient utilization, immune function, and intestinal health of pigs [5,6,7,8,9]. Previous research on the replacement of antibiotics by LAB mainly focused on the impact on immune function [10]. Few studies have focused on the effects of LAB on antioxidant indicators [11,12]. However, pigs are faced with environmental, nutrition, and weaning pressures at all stages of growth at present [13]. The proportion of economic loss caused by weaning stress syndrome has become the main loss in the breeding process. Oxidative stress impairs tissue function, which may be an important cause of reduced growth performance and economic losses in weaning stress syndrome [14]. Therefore, research on improving piglets’ antioxidant capacity is valuable.

*Lactobacillus johnsonii* RS-7, one type of LAB, was isolated from pig feces by the self-built method in our laboratory. We wanted to pay more attention to the effect of *Lactobacillus johnsonii* RS-7 on the antioxidant index of weaned piglets as we observed similar effects in a previous pilot experiment. There was no report about the effect of *Lactobacillus johnsonii* RS-7 on the antioxidant performance of weaned piglets. Therefore, this experiment explored the effects of dietary supplementation of *Lactobacillus johnsonii* RS-7 on the immune function and antioxidant capacity of weaned piglets.

## 2. Materials and Methods

### 2.1. Experiment Design and Animals

A total of one hundred and twenty-eight Duroc × Landrace × Yorkshire piglets weaned at 28 d with an initial body weight (BW) of 8.95 ± 1.15 kg were randomly divided into four treatment groups according to BW and sex. There were eight replicates per treatment group and four weaned piglets (two barrows and two gilts) per replicate (pen). The four dietary treatments were basal diet (CON), and CON with 0.05% (LJ0.05), 0.1% (LJ0.1), and 0.2% (LJ0.2) *Lactobacillus johnsonii* RS-7, respectively. The concentration of *Lactobacillus johnsonii* RS-7 used in this study was 1 × 10^7^ cfu/g. This strain was identified after the laboratory independently extracted it from pig feces. Diet nutrient requirements are configured according to national research council (2012) recommendations and diet in powder form (Table 1) [15]. Each treatment group was fed ad libitum for 28 days. Pigs had free access to water at all times. There was a plastic leaky floor and a separate chute and automatic nipple drinker in each pen. The relative humidity in the pig house was controlled at 50–60%. The temperature of the pig house was gradually reduced from 28 °C to 23 °C.

### 2.2. Growth Performance and Sample Collection

BW was measured at 8 am on days 1, 14, and 28. Feed intake was recorded in pens every day during the experiment period. At 7:00 am on days 14 and 28, one pig was randomly selected from each pen (male to female ratio in each treatment was 1:1), and blood was collected from the portal vein precaval and placed in a vacuum blood collection tube without anticoagulant. Blood samples were centrifuged at 4 °C, 3500× *g* for 10 min to obtain serum for the determination of immune parameters and antioxidant status. After the BW was measured on day 28, one piglet (total 32) was selected from each pen (the ratio of male to female in each treatment was 1:1) was slaughtered and the mucosa of the duodenum, jejunum, and ileum and spleen, liver, and pancreas samples were collected and temporarily stored in liquid nitrogen and then transferred to −80 °C for storage to measure antioxidant status.

### 2.3. Immune Function

The total protein (TP), albumin (ALB), immunoglobulin G (IgG), and immunoglobulin M (IgM) contents in serum were determined by using an automatic biochemical analyzer (Hitachi 7160, Tokyo, Japan). Use an ELISA kit for immunoglobulin A (IgA) (eBioScience, San Diego, CA, USA) to detect the IgA content in serum according to the instructions. Use a microplate reader to measure the absorbance at 450 nm and calculate the IgA concentration through the standard curve.

### 2.4. Antioxidant Index

TP, glutathione (GSH), catalase (CAT), total antioxidant capacity (T-AOC), malondialdehyde (MDA), and total superoxide dismutase (T-SOD) indicators in serum and tissue were measured by spectrophotometer. All tissue samples (the mucosa of the duodenum, jejunum, and ileum, and spleen, liver, pancreas) were homogenized with cold saline at a ratio of 1:10 (*w*:*v*) in a glass homogenizer. All kits used were purchased from Nanjing Jiancheng Bioengineering Institute (Nanjing, China). All samples were counted on a spectrophotometer after being operated according to the instructions of the kit. The total protein assay kit (with standard: BCA method) (A045-3-1) was used for the determination of TP content at 562 nm. A GSH assay kit (spectrophotometric method) (A005-1-2) was used for the determination of GSH content at 420 nm. A catalase (CAT) assay kit (Visible light) (A007-1-1) was used for the determination of CAT content at 405 nm. A total antioxidant capacity assay kit (A015-1-2) was used for the determination of T-AOC content at 520 nm. A malondialdehyde (MDA) assay kit (TBA method) (A003-1-1) was used for the determination of MDA content at 532 nm.

### 2.5. Statistical Analysis

Data were analyzed by ANOVA using the GLM procedure of SAS 9.4 for a randomized complete block design evaluating the level of *Lactobacillus johnsonii* RS-7 added to the diet. The dose-response effect of dietary *Lactobacillus johnsonii* RS-7 was computed using orthogonal polynomial contrasts to evaluate linear and quadratic effects. A post-hoc test was used to compare the control group (0% *Lactobacillus johnsonii* RS-7) versus *Lactobacillus johnsonii* RS-7 added to the diet. For all response criteria, the pen served as the experimental unit. *p* < 0.05 was considered statistically significant.

## 3. Results

### 3.1. Growth Performance

There were linear increases in final body weight (FBW, day 28), average daily feed intake (ADFI) (day 15–28 and day 1–28) and linear and quadratic increases in average daily weight gain (ADG) (day 15–28 and day 1–28) as the amounts of dietary *Lactobacillus johnsonii* RS-7 increased (Table 2; *p* < 0.05). Compared to the CON and LJ0.5, the FBW was higher when LJ0.1 was added (*p* < 0.05). Linear decreases in F:G (Day 15–28 and Day 1–28) were present as the amounts of dietary *Lactobacillus johnsonii* RS-7 increased (*p* < 0.05), and the lowest F:G was found when LJ0.1 was added to the diet. 

### 3.2. Immune Function

The addition of *Lactobacillus johnsonii* RS-7 to the diet increased the concentrations of TP, ALB, IgA, and IgM on day 14 and IgG, IgA, and IgM on day 28 (*p* < 0.05) in the blood, with trait values greater for pigs fed LJ0.1 than CON pigs (Table 3; *p*< 0.05). There was a linear (*p* < 0.05) increase but no quadratic change in the above indicators as the amounts of dietary *Lactobacillus johnsonii* RS-7 increased.

### 3.3. Antioxidants in Serum

On day 28, concentrations of antioxidants (CAT, T-AOC, MDA, T-SOD, and GSH) in serum increased (*p* < 0.05) with the addition of dietary *Lactobacillus johnsonii* RS-7, along with a linear increase (*p* < 0.05) as the amount of dietary *Lactobacillus johnsonii* RS-7 increased. When LJ0.1 was added to the diet, the concentrations of CAT and T-SOD in serum were closer to the maximum (Table 4). With the increase in the amount of *Lactobacillus johnsonii* RS-7 added, the antioxidant indexes in the serum did not show quadratic changes (*p* > 0.05).

### 3.4. Antioxidants in the Intestinal Mucosa

There were linear increases in the concentrations of CAT, T-AOC, T-SOD, and GSH and a linear decrease in the concentration of MDA in intestinal mucosa as the amounts of dietary *Lactobacillus johnsonii* RS-7 increased (Table 5; *p* < 0.05). In duodenal mucosa, the addition of LJ0.1 increased the concentrations of T-AOC and GSH compared to CON (*p* < 0.05). When LJ0.1 or LJ0.2 was added to the diet, the concentrations of T-AOC in jejunal mucosa and CAT, T-SOD, and GSH in ileal mucosa were higher than those of CON (*p* < 0.05), while the concentration of MDA was lower than that of CON (*p* < 0.05). There was a quadratic decrease in the concentrations of MDA in the ileal mucosa as the amounts of dietary *Lactobacillus johnsonii* RS-7 increased (*p* < 0.05).

### 3.5. Antioxidants in the Spleen, Liver, and Pancreas

There were linear increases in the concentrations of CAT, T-AOC, T-SOD, and GSH and linear decreases in the concentration of MDA in the spleen, liver, and pancreas as the amounts of dietary *Lactobacillus johnsonii* RS-7 increased (Table 6; *p* < 0.05). Concentrations of CAT, T-AOC, T-SOD, and GSH in the spleen and liver, T-SOD, and GSH in the pancreas further increased when the dietary inclusion rate for *Lactobacillus johnsonii* RS-7 increased from 0% to 0.2%. When LJ0.1 was added to the diet, the concentration of T-AOC in the pancreas was higher than that of CON (*p* < 0.05). With the increase in the amount of *Lactobacillus johnsonii* RS-7 added, the antioxidant indexes in the spleen, liver, and pancreas did not show quadratic changes (*p* > 0.05).

## 4. Discussion

Weaning stress syndrome is a major challenge for piglets during weaning which may trigger an imbalance in gut health, damage tissues, cause diarrhea, and reduce growth performance [13,16]. It was previously found that oxidative stress caused by weaning stress syndrome may be the main reason for the reduction of economic benefits because the excessive oxidative free radicals produced by it can damage deoxyribonucleic acid (DNA) and protein and tissue function [17]. So, it is very important to improve the antioxidant status of piglets and relieve oxidative stress. Previous studies have shown that adding probiotics can effectively improve the antioxidant status of pigs or other animals [12,18,19,20,21]. However, the effect of *Lactobacillus johnsonii* RS-7 on the antioxidant status of weaned piglets has not been reported. We studied the growth performance, immune function, and antioxidant status of weaned piglets by adding *Lactobacillus johnsonii* RS-7 to the diet and found that it can effectively improve growth performance (ADFI and ADG), enhance immune function, and improve antioxidant status.

Growth performance is an important economic indicator. The growth performance of pigs is affected by factors such as the environment of the pig house, feeding conditions, and nutritional composition of the diet [22,23,24]. This study found linear increases in FBW (day 28), ADFI (day 15–28 and day 1–28), and ADG (day 15–28 and day 1–28) as the amounts of dietary *Lactobacillus johnsonii* RS-7 increased. Similar results that probiotics and LAB improved growth performance have been found in other studies [25,26,27]. Feeding compound probiotics (*Bacillus subtilis* endospore and *Clostridium butyricum* endospore complex) can increase the ADG and G: F of growing and finishing pigs effectively. Feeding *Lactobacillus* complex (*Lactobacillus reuteri* ZJ625, *Lactobacillus reuteri* VB4, *Lactobacillus salivarius* ZJ614, and *Streptococcus salivarius* NBRC13956) to weaned piglets can improve ADG and feed conversion ratio effectively. Feeding compound probiotics (*Lactobacillus plantarum*, *Lactobacillus fermentum*, and *Enterococcus faecium*) to weaned piglets can increase ADG and FBW effectively. In addition, the current study also found that feeding *Lactobacillus johnsonii* RS-7 did not affect the growth performance of weaned piglets during the period days 0–14. This was probably because the replacement of intestinal flora was a long process, which required a long-term supply of corresponding probiotics to exert its effect. Our experimental results also showed that probiotics were more effective during days 15–28. The LJ0.1 group had higher FBW and ADG and lower F:G compared with the control group or LJ0.05. In addition, although ADG and F:G from days 15 to 28 changed linearly and quadratically, the index of LJ0.1 was better than that of LJ0.2. Likewise, we observed similar results for immune function and antioxidant indices. The above results indicated that the excessive addition of *Lactobacillus johnsonii* RS-7 did not lead to better growth performance.

Immunoglobulin mainly includes IgA, IgM, IgG, immunoglobulin E (IgE), and Immunoglobulin D (IgD), among which IgA is mainly divided into serotype and secretory immunoglobulin A (SIgA), which can reduce the adhesion rate of pathogenic bacteria, anti-inflammation, and anti-infection; IgG has the functions of antibacterial, antiviral, and immune regulation; IgE is related to allergy, and has a high affinity for basophils and mast cells [28,29]. After weaning, piglets were affected by weaning stress, which destroyed the balance of the original intestinal flora and reduced the immune function of piglets. Preliminary studies have shown that adding *Lactobacillus fermentum* I5007 to the diet can significantly increase the content of IgM, IgG, and IgA in the blood of piglets, and supplementation of *Lactobacillus plantarum* and fructooligosaccharides in weaned piglets’ diet can significantly increase serum IgG and IgA levels. Our study also found that the addition of *Lactobacillus johnsonii* RS-7 (CON vs. LAB) to the diet increased the concentrations of TP, ALB, IgA and IgM on day 14 and IgG, IgA, and IgM on day 28. In addition, as the amount of *Lactobacillus johnsonii* RS-7 increased, the concentration of TP, ALB, IgA, and IgM on day 14 and IgG, IgA, and IgM on day 28 showed a linear change without a quadratic change. The results showed that the indicators of linear change in the LJ0.1 group were better than those in other treatment groups. In addition, from our results, it can be seen that the immune function of weaned piglets was improved by day 14, but no improvement in growth performance was shown. The reason for the above may be that the positive effect of immune repair may repair the effects of weaning stress to a certain extent, but it has not achieved a significant effect. Therefore, it was shown that dietary supplementation of *Lactobacillus johnsonii* RS-7 can improve the immune status of piglets and prevent oxidative stress.

Reactive oxygen species (ROS) and reactive nitrogen species (RNS) lead to oxidative stress, which in turn produces tissue function damage [14]. In the enzymatic antioxidant system, superoxide dismutase and glutathione peroxidase can eliminate excessive oxidative free radicals through biochemical reactions to achieve detoxification [17,30]. SOD and GSH work together to detoxify superoxide anion and hydrogen peroxide in cells [30]. CAT performs the same function as GSH. T-AOC can reflect the ability of a non-enzymatic antioxidant defense system [31]. MDA, the main product of lipid peroxidation, reflects oxidative stress levels [30]. In our study, we collected serum, intestinal mucosa, spleen, liver, and pancreas samples and analyzed the contents of CAT, T-AOC, T-SOD, GSH, and MDA. In the serum samples, we found that the levels of CAT, T-AOC, TSOD, and GSH increased linearly and the levels of MDA decreased linearly with the increase of *Lactobacillus johnsonii* RS-7 content in the diet. In addition, similar trends to the above results were also found in intestinal mucosa and visceral tissues. In studies in sows and lambs, an increase in TAOC was also found with the addition of probiotics [32,33]. The above results show that the addition of *Lactobacillus johnsonii* RS-7 can improve the antioxidant status of the whole body, reduce lipid peroxidation, and enhance the responsiveness of the antioxidant defense system effectively.

## 5. Conclusions

The current research has proved that dietary supplementation of *Lactobacillus johnsonii* RS-7 can increase the ADFI and ADG of weaned piglets and improve the F:G effectively. In addition, adding *Lactobacillus johnsonii* RS-7 to the diet can also enhance the immune function and antioxidant status of weaned pigs. Specifically, 0.1% (1 × 10^7^ cfu/g) was recommended as the optimal level.

## Figures and Tables

**Table 1:** Basal diet composition and nutritional level.

Ingredients, g/kg	
Extruded corn	23.20
Wheat	24.00
Extruded soybean (38% CP)	14.00
Soybean meal (42% CP)	5.00
Spray-dried whey (12% CP)	7.00
Fish meal (59% CP)	4.50
Soya protein concentrate (65% CP)	5.00
Soya oil	4.00
Limestone	0.60
Monocalcium phosphate	0.60
L-Lys·HCl	0.20
Nacl	0.30
Choline chloride	0.10
Vitamin premix ^a^	0.50
Mineral premix ^b^	1.00
Total	100.00
Calculated nutrients, %	
ME, MJ/kg	14.15
CP	19.90
Total Lys	1.43
Total Met	0.39
Ca	0.77
Available P	0.46

^a^ Provided per kg of mixed diet: vitamin A, 12,000 IU/kg; vitamin D3, 3,200 IU/kg; vitamin K3, 2.5 mg; vitamin E, 80 mg; vitamin B1, 2.5 mg; vitamin B2, 6.5 mg; vitamin B6, 5 mg; vitamin B12, 0.05 mg; niacin, 45 mg; and D-pantothenic acid, 20 mg. ^b^ Provided per kg of mixed diet: folic acid, 1.5 mg; biotin, 0.15 mg; Fe, 150 mg as ferrous sulfate; Cu, 125 mg as copper sulfate; Zn, 200 mg as zinc oxide; Mn, 30 mg as manganous oxide; I, 0.3 mg as potassium iodide; and Se, 0.3 mg as selenium selenite.

**Table 2:** Effects of dietary supplementation of *Lactobacillus johnsonii* RS-7 on growth performance of weaned piglets.

Items	CON	LJ0.05	LJ0.1	LJ0.2	SEM	*p*-Value		
						CON vs. LJ	Linear	Quadratic
Initial body weight, kg	9.00	8.93	8.94	8.94	0.240	0.805	0.855	0.883
Day 14 body weight, kg	14.21	14.26	14.48	14.35	0.316	0.675	0.562	0.835
Day 28 body weight, kg	19.94 ^b^	20.04 ^b^	21.29 ^a^	21.11 ^ab^	0.344	0.052	0.010	0.183
Average daily feed intake, g								
Day 1–14	533.2	559.3	560.6	549.9	15.4	0.186	0.220	0.517
Day 15–28	619.6	626.6	690.1	697.8	20.6	0.053	0.022	0.270
Day 1–28	576.4	592.9	625.5	623.9	12.8	0.020	0.012	0.616
Average daily weight gain, g								
Day 1–14	372.3	381.3	395.5	386.6	8.48	0.121	0.063	0.798
Day 15–28	408.9 ^b^	412.5 ^b^	486.6 ^a^	483.0 ^a^	12.4	0.009	0.001	0.027
Day 1–28	390.6 ^b^	396.9 ^b^	441.1 ^a^	434.8 ^a^	6.49	0.002	0.001	0.024
Feed to gain ratio								
Day 1–14	1.43	1.47	1.42	1.42	0.031	0.918	0.804	0.304
Day 15–28	1.52 ^a^	1.52 ^a^	1.42 ^b^	1.44 ^ab^	0.023	0.076	0.007	0.086
Day 1–28	1.48 ^ab^	1.49 ^a^	1.42 ^b^	1.43 ^ab^	0.017	0.240	0.025	0.032

Note: CON, basal diet; LJ0.05, LJ0.1, and LJ0.2, add 0.05% (LJ0.05), 0.1% (LJ0.1), and 0.2% (LJ0.2) *Lactobacillus johnsonii* RS-7 to the basal diet. ^a,b^ Means in the same row with different superscripts differ significantly (*p* < 0.05).

**Table 3:** Effects of dietary supplementation of *Lactobacillus johnsonii* RS-7 on immune function of weaned piglets.

Items, g/L	CON	LJ0.05	LJ0.1	LJ0.2	SEM	*p*-Value		
						CON vs. LJ	Linear	Quadratic
Day 14								
TP	50.55 ^b^	52.60 ^b^	55.28 ^a^	55.35 ^a^	0.653	0.001	0.001	0.698
ALB	26.84	28.41	29.05	29.13	0.658	0.011	0.025	0.566
IgG	7.44	8.13	8.48	8.45	0.410	0.057	0.085	0.739
IgA	1.19 ^b^	1.26 ^b^	1.66 ^a^	1.54 ^ab^	0.094	0.019	0.001	0.170
IgM	0.74 ^b^	0.83 ^ab^	1.02 ^a^	0.93 ^ab^	0.056	0.010	0.001	0.458
Day 28								
TP	55.99	56.19	58.58	58.96	0.968	0.119	0.069	0.323
ALB	24.65	25.36	26.40	26.34	0.665	0.079	0.073	0.843
IgG	7.36	7.64	8.79	8.38	0.363	0.048	0.010	0.333
IgA	1.02 ^b^	1.13 ^ab^	1.27 ^a^	1.26 ^a^	0.056	0.005	0.004	0.878
IgM	0.77 ^b^	0.88 ^ab^	1.12 ^a^	1.13 ^a^	0.074	0.005	0.002	0516

Note: CON, basal diet; LJ0.05, LJ0.1, and LJ0.2, add 0.05% (LJ0.05), 0.1% (LJ0.1), and 0.2% (LJ0.2) *Lactobacillus johnsonii* RS-7 to the basal diet; TP, total protein; ALB, albumin; IgG, immunoglobulin G; IgM, immunoglobulin M; IgA, immunoglobulin A. ^a,b^ Means in the same row with different superscripts differ significantly (*p* < 0.05).

**Table 4:** Effects of dietary supplementation of *Lactobacillus johnsonii* RS-7 on antioxidant in serum of weaned piglets.

Items	CON	LJ0.05	LJ0.1	LJ0.2	SEM	*p*-Value		
						CON vs. LJ	Linear	Quadratic
Day 14								
CAT, U/mL	18.64	19.58	20.83	20.55	0.638	0.030	0.022	0.843
T-AOC, U/mL	2.50	2.63	3.01	2.98	0.166	0.068	0.038	0.524
MDA, nmol/mL	4.36	4.17	2.84	3.15	0.408	0.059	0.012	0.249
T-SOD, U/mL	17.75	18.25	18.28	18.21	0.392	0.267	0.353	0.626
GSH, mgGSH/L	3.71	4.06	4.36	4.33	0.421	0.264	0.284	0.962
Day 28								
CAT, U/mL	16.95 ^b^	17.14 ^ab^	18.73 ^a^	18.60 ^ab^	0.439	0.039	0.008	0.204
T-AOC, U/mL	2.35	2.63	3.09	3.10	0.214	0.027	0.022	0.723
MDA, nmol/mL	4.13	3.71	2.92	2.98	0.339	0.028	0.018	0.659
T-SOD, U/mL	16.16 ^b^	16.49 ^b^	17.96 ^a^	17.29 ^ab^	0.362	0.027	0.002	0.201
GSH, mgGSH/L	3.63	4.11	4.94	4.76	0.353	0.025	0.014	0.700

Note: CON, basal diet; LJ0.05, LJ0.1, and LJ0.2, add 0.05% (LJ0.05), 0.1% (LJ0.1), and 0.2% (LJ0.2) *Lactobacillus johnsonii* RS-7 to the basal diet; CAT, catalase; T-AOC, total antioxidant capacity; MDA, malondialdehyde; T-SOD, total superoxide dismutase; GSH, glutathione. ^a,b^ Means in the same row with different superscripts differ significantly (*p* < 0.05).

**Table 5:** Effects of dietary supplementation of *Lactobacillus johnsonii* RS-7 on antioxidant in the intestinal mucosa of weaned piglets.

Items	CON	LJ0.05	LJ0.1	LJ0.2	SEM	*p*-Value		
						CON vs. LJ	Linear	Quadratic
Duodenal mucosa								
CAT, U/gprot	14.14	14.80	18.05	17.83	1.32	0.088	0.045	0.430
T-AOC, U/mgprot	0.24 ^b^	0.26 ^ab^	0.33 ^a^	0.32 ^ab^	0.020	0.028	0.005	0.232
MDA, nmol/mL	33.25	30.88	20.68	19.80	3.77	0.047	0.018	0.354
T-SOD, U/mL	21.75	23.00	29.38	29.13	1.99	0.036	0.012	0.302
GSH, mgGSH/gprot	5.41 ^b^	5.63 ^ab^	10.18 ^a^	9.93 ^ab^	1.18	0.046	0.008	0.145
Jejunal mucosa								
CAT, U/gprot	4.13	4.25	6.49	6.41	0.664	0.065	0.018	0.205
T-AOC, U/mgprot	0.25 ^b^	0.25 ^b^	0.33 ^a^	0.34 ^a^	0.021	0.037	0.008	0.141
MDA, nmol/mL	34.25	34.63	20.25	19.88	3.88	0.072	0.016	0.132
T-SOD, U/mL	12.21	13.91	17.50	17.93	1.55	0.030	0.023	0.624
GSH, mgGSH/gprot	1.85	1.93	2.85	2.86	0.315	0.082	0.033	0.280
Ileal mucosa								
CAT, U/gprot	5.55 ^c^	5.69 ^bc^	8.01 ^ab^	8.11 ^a^	0.609	0.038	0.008	0.154
T-AOC, U/mgprot	0.13	0.15	0.21	0.21	0.021	0.034	0.019	0.508
MDA, nmol/mL	88.25 ^a^	90.63 ^a^	60.75 ^b^	62.25 ^b^	5.63	0.043	0.002	0.027
T-SOD, U/mL	8.61 ^b^	8.65 ^b^	10.38 ^a^	10.51 ^a^	0.418	0.035	0.006	0.110
GSH, mgGSH/gprot	1.13 ^b^	1.31 ^ab^	1.86 ^a^	1.96 ^a^	0.173	0.012	0.005	0.400

Note: CON, basal diet; LJ0.05, LJ0.1, and LJ0.2, add 0.05% (LJ0.05), 0.1% (LJ0.1), and 0.2% (LJ0.2) *Lactobacillus johnsonii* RS-7 to the basal diet; CAT, catalase; T-AOC, total antioxidant capacity; MDA, malondialdehyde; T-SOD, total superoxide dismutase; GSH, glutathione; prot, protein. ^a,b,c^ Means in the same row with different superscripts differ significantly (*p* < 0.05).

**Table 6:** Effects of dietary supplementation of *Lactobacillus johnsonii* RS-7 on antioxidant in spleen, liver, and pancreas of weaned piglets.

Items	CON	LJ0.05	LJ0.1	LJ0.2	SEM	*p*-Value		
						CON vs. LJ	Linear	Quadratic
Spleen								
CAT, U/gprot	11.13 ^b^	12.09 ^ab^	15.26 ^a^	15.2 ^a^	0.823	0.008	0.001	0.282
T-AOC, U/mgprot	0.26 ^b^	0.25 ^b^	0.35 ^a^	0.35 ^a^	0.023	0.059	0.007	0.072
MDA, nmol/mL	154.9	150.0	123.4	125.0	8.79	0.049	0.017	0.321
T-SOD, U/mL	260.6 ^b^	264.3 ^ab^	297.4 ^ab^	302.1 ^a^	10.0	0.042	0.016	0.244
GSH, mgGSH/gprot	1.75 ^b^	1.85 ^ab^	2.78 ^a^	2.83 ^a^	0.954	0.035	0.009	0.202
Liver								
CAT, U/gprot	201.3 ^b^	205.1 ^ab^	245.9 ^a^	246.3 ^a^	11.2	0.038	0.009	0.190
T-AOC, U/mgprot	0.59 ^b^	0.61 ^b^	0.93 ^a^	0.96 ^a^	0.081	0.034	0.007	0.139
MDA, nmol/mL	297.0	276.9	202.9	204.1	27.9	0.049	0.024	0.437
T-SOD, U/mL	1150 ^b^	1161 ^b^	1432 ^ab^	1485 ^a^	76.0	0.044	0.014	0.174
GSH, mgGSH/gprot	1.89 ^b^	1.89 ^b^	3.28 ^a^	3.62 ^a^	0.312	0.027	0.004	0.074
Pancreas								
CAT, U/gprot	5.01	5.28	5.79	5.68	0.255	0.065	0.040	0.692
T-AOC, U/mgprot	0.15 ^b^	0.20 ^ab^	0.26 ^a^	0.21 ^ab^	0.016	0.002	0.001	0.829
MDA, nmol/mL	47.8	47.5	29.1	26.6	5.48	0.066	0.023	0.188
T-SOD, U/mL	242.1 ^b^	250.3 ^b^	296.0 ^a^	310.9 ^a^	11.8	0.012	0.003	0.204
GSH, mgGSH/gprot	2.33 ^b^	2.50 ^ab^	3.19 ^ab^	3.29 ^a^	0.249	0.037	0.021	0.407

Note: CON, basal diet; LJ0.05, LJ0.1, and LJ0.2, add 0.05% (LJ0.05), 0.1% (LJ0.1), and 0.2% (LJ0.2) *Lactobacillus johnsonii* RS-7 to the basal diet; CAT, catalase; T-AOC, total antioxidant capacity; MDA, malondialdehyde; T-SOD, total superoxide dismutase; GSH, glutathione prot, protein. ^a,b^ Means in the same row with different superscripts differ significantly (*p* < 0.05).

## Data Availability

Data will be made available upon reasonable request.
